# 

**Published:** 1885-10

**Authors:** 


This Half Page for Sale.—The last to be had of "prefered space." Price $50 a year.

---

### NOTICE.

Others than subscribers, receiving this Journal, will please keep the copies sent, and notify us on a postal card if *they do not wish* to subscribe. Silence gives consent, and the Journal will be continued to those who do not countermand it, and such will be considered subscribers and asked to pay the subscription at their earliest convenience.

---

Get Us Up Clubs.—The sender of six subscribers and $12 dollars will receive the Journal one year and and a $5 Medical Work just out of press. Five subscribers and $10, a $4 book and the Journal one year.

---

Premium for Back Numbers.—Twenty-five cents each will be paid for copies of our July, August and September numbers in good order; or your subscription will be advanced one month for every copy sent, of either issue.

